# Intravascular papillary endothelial hyperplasia following adipose-derived mesenchymal stem cell implantation: A case report

**DOI:** 10.1016/j.jdcr.2026.01.012

**Published:** 2026-01-19

**Authors:** Sharon Dhaliwal, Zaina Rashid, Hongyu Yang

**Affiliations:** a Midwestern University Arizona College of Osteopathic Medicine, Glendale, Arizona; b La Peau Dermatology, Mesa, Arizona; c Tri-State Pathology Associates, St Vincent Evansville Hospital, Evansville, Indiana

**Keywords:** intravascular papillary endothelial hyperplasia (IPEH), Masson’s tumor, Mesenchymal stem cells (MSC)

## Introduction

Intravascular papillary endothelial hyperplasia (IPEH), also known as Masson’s tumor, is a rare, benign vascular lesion of the skin and subcutaneous tissue characterized by reactive endothelial cell proliferation, often associated with thrombus formation, vascular injury, trauma, or malformations.^1^, ^2^, ^3^ Typically presenting as a slowly growing, red or blue mass in the head, neck, upper extremities, or lower extremities, IPEH may be asymptomatic or tender.^1^^,^^2^^,^^4^ To our knowledge, no published reports have described IPEH following mesenchymal stem cell (MSC) implantation. Adipose-derived MSCs have procoagulant properties that may promote local thrombus formation, potentially contributing to IPEH development.^5^ We present a novel case of IPEH occurring 5 months after adipose-derived MSC injection and propose a potential mechanism.

## Case report

**Fig 1:**
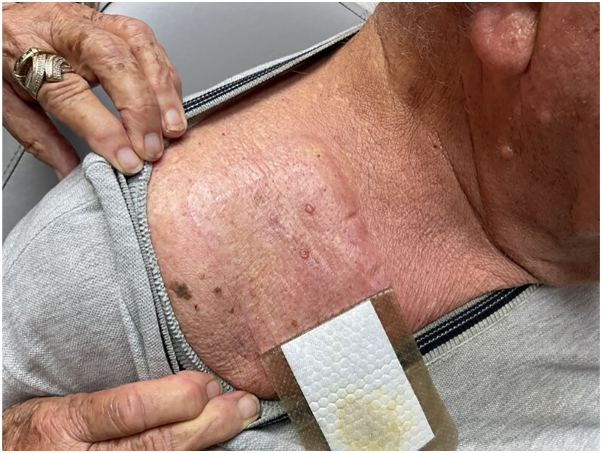
Two 8 mm, *flesh-colored*, *dome-shaped papules* in the patient’s right supraclavicular region with active serosanguinous drainage.

**Fig 2:**
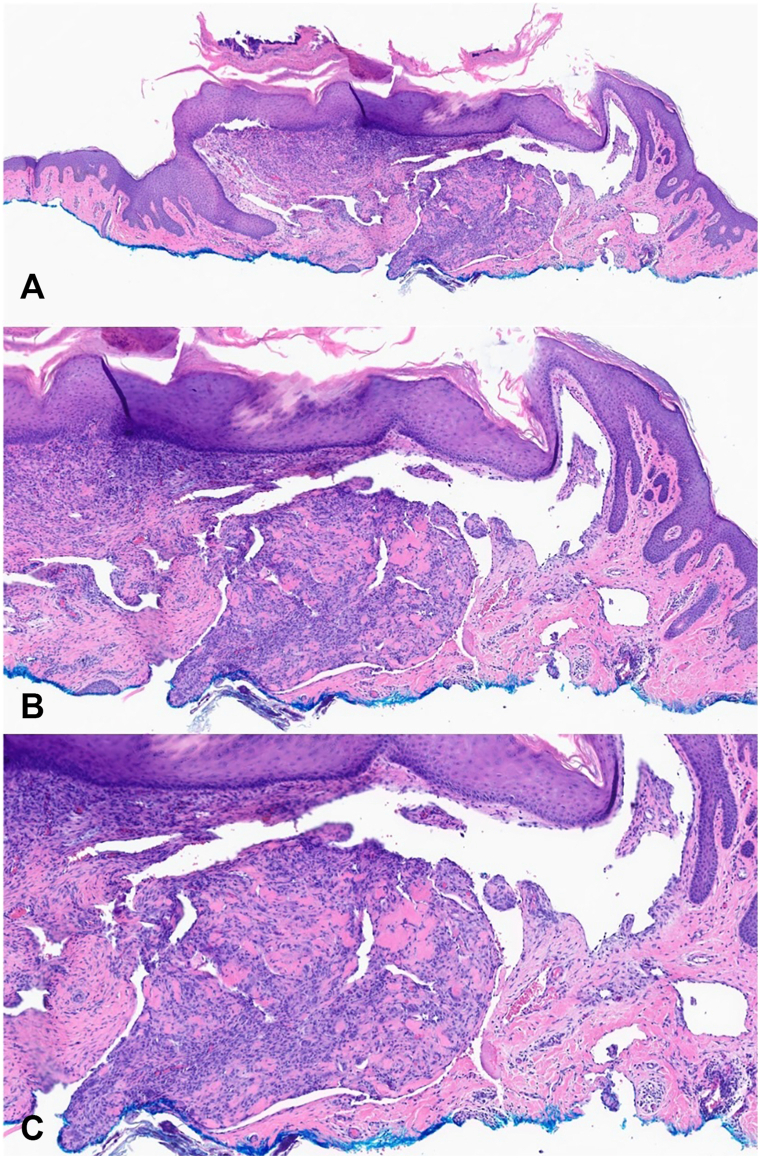
Histopathological images of the lesional biopsy at **(A)** 10 × , **(B)** 20 × , and **(C)** 40 × magnification, showing a well-circumscribed intravascular lesion with papillary endothelial proliferation, plump endothelial cells, and no cytologic atypia, consistent with IPEH. *IPEH*, Intravascular papillary endothelial hyperplasia.

An 85-year-old man with hypercholesterolemia presented to the dermatology clinic with two 8 mm, flesh-colored, dome-shaped papules in the right supraclavicular region, both with active yellow serosanguineous drainage, persisting for 8 months (Fig 1). The patient reported receiving adipose-derived MSC injections in April 2024 for adhesive capsulitis and a prior rotator cuff injury, administered in the same supraclavicular region, 5 months before the papules appeared. In November 2024, his primary care provider diagnosed a presumed infected cyst and prescribed oral antibiotics. Despite no improvement after 2 weeks, the patient applied bandages and delayed further evaluation until presenting to dermatology clinic. Biopsy of the papules and bacterial cultures of the drainage were performed to rule out infection. Histopathology revealed a well-demarcated, polypoid vascular lesion in the superficial dermis with centrally dilated vessels, intravascular proliferation of reactive endothelial cells forming papillary structures lined by plump endothelial cells, and focal stromal fibrosis (Fig 2). No cytologic atypia, tumor necrosis, or infiltrative growth was observed, these findings are consistent with IPEH. Immunohistochemistry was not performed as the diagnosis was established based on characteristic morphological features on routine histopathology. Bacterial cultures showed no growth. Complete excision of the papules resulted in full recovery with no recurrence or drainage at 4-month follow-up.

## Discussion

IPEH accounts for 2% to 4% of vascular tumors of the skin and soft tissues.^1^ It may mimic other benign (eg, cherry hemangioma, pyogenic granuloma) or malignant (eg, angiosarcoma, Kaposi’s sarcoma) vascular tumors, necessitating accurate diagnosis to avoid overly aggressive treatment.^6^^,^^7^ In a recent multicenter cohort study of 261 patients, IPEH was rarely considered clinically, with lesions most often mistaken for cysts, hemangiomas, or lipomas, highlighting the importance of correlating clinical and histopathological findings for accurate diagnosis.^4^ Histopathology, the gold standard for diagnosis, reveals papillary endothelial proliferation within a dilated vascular lumen, associated with an organizing thrombus, without significant mitotic activity, atypia, or necrosis.^3^^,^^6^^,^^7^ IPEH typically stains positive for CD31, CD34, and factor VIII-related antigen and negative for CD105.^6^ IPEH is often linked to thrombus-forming events, such as vascular trauma, stasis, or malformations.^3^^,^^6^ Prior reports describe IPEH following repeated trauma^1^ or needle-stick injury,^8^ with basic fibroblast growth factor from macrophages driving endothelial proliferation.^9^

Our case, involving an 85-year-old man with IPEH 5 months after adipose-derived MSC injections in the right supraclavicular region, is the first reported association with MSC therapy. Adipose-derived MSCs express high levels of tissue factor, activating the extrinsic coagulation pathway and promoting thrombus formation.^5^^,^^10^ This procoagulant activity, combined with injection-related trauma, likely initiated a local thrombus, serving as a nidus for IPEH. The patient’s hypercholesterolemia may have further predisposed him to thrombosis, while reduced mobility secondary to adhesive capsulitis could have exacerbated vascular stasis, together creating a favorable environment for venous thrombosis and subsequent IPEH development. The atypical presentation of papules with chronic serosanguinous drainage expands the clinical spectrum of IPEH and underscores the importance of clinician awareness to ensure accurate diagnosis and management. Complete surgical excision, as performed in this patient, is curative, with recurrence rare unless excision is incomplete.^2^

A limitation of this report is that immunohistochemistry was not performed to further confirm the diagnosis of IPEH. The diagnosis was established based on characteristic morphologic features on routine histopathology, including the absence of cytologic atypia, necrosis, or infiltrative growth. Although immunohistochemistry could have provided additional diagnostic confirmation and exclusion of alternative vascular pathologies, the overall histopathological findings strongly support the diagnosis. Overall, clinicians should be aware of the potential thrombotic risks associated with adipose-derived MSC injections, particularly in patients with predisposing risk factors. Further studies are needed to assess the incidence of vascular complications following MSC therapy and to clarify patient and procedure related risk factors.

## Conflicts of interest

None disclosed.
